# Cortical Dynamics of Semantic Processing during Sentence Comprehension: Evidence from Event-Related Optical Signals

**DOI:** 10.1371/journal.pone.0070671

**Published:** 2013-08-01

**Authors:** Jian Huang, Suiping Wang, Shiwei Jia, Deyuan Mo, Hsuan-Chih Chen

**Affiliations:** 1 Center for Studies of Psychological Application and School of Psychology, South China Normal University, Guangzhou, China; 2 Key Laboratory of Mental Health and Cognitive Science of Guangdong Province, South China Normal University, Guangzhou, China; 3 Department of Psychology, Chinese University of Hong Kong, Shatin, N.T., Hong Kong S.A.R., China; Lancaster University, United Kingdom

## Abstract

Using the event-related optical signal (EROS) technique, this study investigated the dynamics of semantic brain activation during sentence comprehension. Participants read sentences constituent-by-constituent and made a semantic judgment at the end of each sentence. The EROSs were recorded simultaneously with ERPs and time-locked to expected or unexpected sentence-final target words. The unexpected words evoked a larger N400 and a late positivity than the expected ones. Critically, the EROS results revealed activations first in the left posterior middle temporal gyrus (LpMTG) between 128 and 192 ms, then in the left anterior inferior frontal gyrus (LaIFG), the left middle frontal gyrus (LMFG), and the LpMTG in the N400 time window, and finally in the left posterior inferior frontal gyrus (LpIFG) between 832 and 864 ms. Also, expected words elicited greater activation than unexpected words in the left anterior temporal lobe (LATL) between 192 and 256 ms. These results suggest that the early lexical-semantic retrieval reflected by the LpMTG activation is followed by two different semantic integration processes: a relatively rapid and transient integration in the LATL and a relatively slow but enduring integration in the LaIFG/LMFG and the LpMTG. The late activation in the LpIFG, however, may reflect cognitive control.

## Introduction

Semantic processing during sentence comprehension involves retrieving the meanings of individual words and integrating those meanings into a larger semantic unit [1]–[4]. However, the temporal and spatial dynamics of the neural mechanisms underlying these semantic processes remain unclear. For example, there is no consistent evidence for the exact time point at which lexical-semantic retrieval and semantic integration occur in sentence processing. Behavioral and eye movement studies tend to support that these two processes could occur very rapidly, i.e., before 250 ms [5]–[7]. Nevertheless, using the ERP technique, very few studies have shown that these semantic processes could affect early ERP components such as N1 and P2 in sentence processing [8]–[11]. Rather, a large number of studies have consistently shown that various semantic variables could affect a relatively late ERP component, i.e. N400, which leads to the well-known conclusion that semantic processing occurs in the N400 time window typically between 300 to 500ms [12]–[15]. In the spatial domain, studies using techniques with high spatial resolution such as PET and fMRI have consistently shown that the left posterior middle temporal gyrus (LpMTG) is engaged in lexical-semantic retrieval [14], [15], [16]. The question remains, however, as to which brain regions are responsible for semantic integration. Indeed, although previous fMRI studies have frequently shown that the left inferior frontal gyrus (LIFG) plays a significant role in semantic integration [4], [13], [15], other imaging studies on auditory language comprehension [17]–[19] have revealed that the anterior temporal lobe (ATL), especially the LATL, is recruited during semantic integration. Some researchers thus propose that the LATL is responsible for semantic integration and the LIFG is for the general cognitive processes [14], [20]. In other words, the roles of the LIFG and the LATL in semantic integration during language comprehension have not yet been conclusively determined.

Although separately recorded ERP and fMRI data can provide important temporal and spatial information, respectively, about the neural basis of semantic processing, different semantic processes may interact with each other across times, consequently leading to the variability of the activation pattern associated with the same process in different time windows [5], [9], [21], [22]. To clarify the time course and the interaction of different semantic processes more effectively, techniques with both high temporal and spatial resolutions should be considered. The current study thus attempts to explore this issue by using the event-related optical signal (EROS) imaging.

The EROS imaging method uses near-infrared light to identify changes in the light-scattering properties of cortical tissues related to neuronal activity [23]. With its relatively high temporal (less than 100 ms) and spatial resolution (5–10 mm; [24]), this technique allows us to explore the dynamic activation patterns of several brain regions closely related to semantic processing, especially the LATL, the LIFG and the LpMTG, in an attempt to reveal how these brain areas work together to accomplish semantic processing during sentence comprehension. It is worth noting, however, that the EROS technique is relatively new and its methodology is still under development. Tse et al. [25] first used the EROS technique to explore the neural dynamics of semantic and syntactic processing in sentence comprehension. By comparing the processing of semantically incongruent sentences with that of semantically congruent ones, they revealed the dynamics of semantic processing in the left hemisphere. In the time window between 179 and 204 ms, they observed stronger activation in the LS/MTG for incongruent sentences than for congruent ones. In the N400 time window, stronger activation was observed for incongruent sentences in the LS/MTG at approximately 384 ms, followed by stronger activation in the LIFG between 512 and 563 ms. After the N400 time window, the LS/MTG was activated again. These results suggest that semantic integration may be subsequent to lexical-semantic retrieval. Moreover, the LS/MTG and the LIFG may interact during the semantic processing in the N400 time.

A few limitations of the study by Tse et al. [25] should be noted. First, the violation paradigm they used may recruit violation detection and repairing processes that are not part of normal sentence reading [26], [27]. Moreover, the semantic acceptability judgment task in their study requires essentially a “yes” response to the normal sentences and a “no” response to the violated sentences. Different processes that are unrelated to language processing may be recruited during such different responses (i.e., “yes” vs. “no”), confounding the semantic integration difference of interest [28], [29]. More importantly, the montages used in their study covered only some parts of the LATL, which may be the reason why no significant activation was observed in the LATL.

Using the EROS technique and an expectancy paradigm that involved only normal sentences differing in contextual expectancy [30], the current study explored the dynamic brain activations of semantic processing. Following most previous fMRI studies and the only EROS study on this issue, we adopted visual stimuli so that our results could be directly compared with previous ones. Participants were asked to read contextually constraining sentences containing expected or unexpected final words and to perform semantic plausibility judgment on each sentence. Given that both the expected and unexpected sentences were semantically plausible and required the same response pattern, confounding processes such as violation repair were not of concern. Indeed, our previous fMRI study using the same paradigm revealed that sentences with unexpected final words led to stronger activation in some major brain regions associated with semantic processing, such as the LpMTG and the LIFG, suggesting that participants needed to retrieve the semantic meanings of the key words for semantic integration in the “unexpected condition” [31]. This paradigm is thus suitable to be used for investigating the brain dynamics of lexical-semantic retrieval and semantic integration. It is also important to note that we focused on the left hemisphere, like Tse et al. [25], but we improved the montages so that we could detect the left frontal and temporal gyrus, including most parts of the LATL. Furthermore, to make sure that the processes revealed by the EROS data are indeed reflecting the semantic processing, we simultaneously recorded the ERPs elicited by the target word from three electrodes in the midline where the semantic N400 and the late positivity typically maximize.

## Materials and Methods

### Ethics Statement

Written informed consent was obtained from each individual. Approval for this experiment was obtained from the institutional review board of Chinese University of Hong Kong and South China Normal University.

### Participants

Fifteen native Mandarin speakers (11 females) were paid to participate in this experiment. The participants ranged in age from 19 to 25 years (mean = 22.2). All of the participants were right-handed and had normal or corrected-to-normal vision.

### Experimental Materials

The stimuli were prepared by constructing 300 contextually biased sentence stems and giving these to 40 participants who performed a cloze task in which they were instructed to complete each sentence using the first two-character noun that came to mind. A noun was considered to be expected or unexpected if it was generated by more than 75% or fewer than 5% of the participants, respectively. Subsequently, 300 sentence pairs were constructed, each of which comprised 2 versions of the sentence that differed only in whether the target word (the noun) was expected or unexpected.

As shown in Table 1, neither frequencies [32] (*t*_(299)_ = 0.88, *p* = 0.4) nor the numbers of strokes (*t*_(299)_ = 1.51, *p* = 0.13) in the target words differed between the expected vs. unexpected conditions. To ensure that the sentences in these two conditions were semantically congruent, an additional 32 participants from the same subject pool were asked to make semantic plausibility judgments about the sentences. The mean semantic plausibility values for the expected and unexpected conditions were 4.69 and 4.27, respectively, suggesting that all of the sentences we used were plausible.

**Table 1:** Examples and rating results for the two conditions.

Condition	Exemplar	Frequency	Average stroke numbers	Reasonable
Expected	???????????	30.99	16.06	4.69
	Xiaowang go to barber shop to trim hair.	(58.49)	(4.06)	(0.24)
Unexpected	???????????	39.60	15.50	4.27
	Xiaowang go to barber shop to trim mustache.	(72.35)	(4.64)	(0.46)

Notes. The key word in each sentence is underlined. The frequency is given in occurrences per million words. SDs are shown in brackets. For the semantic reasonableness ratings, a score of 5 represents the most plausible, and a score of 1 represents the most implausible.

Two counterbalanced lists were created. Each contained 300 experimental sentences (150 with expected targets and 150 with unexpected targets) and 200 filler sentences that contained semantic violations in order to balance the number of positive and negative responses to a certain extent. In other words, each participant read 500 sentences in total. Within each list, each sentence stem was presented only once, and across the two lists, each sentence stem appeared in all two conditions. Each sentence was divided into several meaning constituents (from 4 to 9 constituents; 6 on average) and the target word always appeared as the final constituent of a sentence.

### Procedures

Participants were randomly assigned to one of the two stimulus lists and were tested individually in a sound-attenuating, electrically shielded booth. Sentences were presented sequentially, one constituent at a time at the center of the screen. Each trial began with a 300-ms presentation of a fixation cross at the center of the screen, after which a blank screen was presented for 200 ms. This initiation sequence was followed by the presentation of the first constituent. Each constituent appeared on the screen for 400 ms, and the inter-stimulus interval (ISI) that elapsed before the presentation of each subsequent constituent was 200 ms in duration. The period that completed the sentence was presented together with the final word that subtended a visual angle around 2°. The participants were instructed to determine whether the sentence was plausible or not and to indicate their choice by pressing the “YES” button or the “NO” button in a response box after the presentation of the sentence. The participants were given 22 practice sentences at the beginning of their test sessions to familiarize them with the procedure. The sentences in a given stimulus list were presented in a random order. Within each session, the 500 sentences were grouped into 20 blocks with 25 sentences in each block. And the participants were given a 10-second-break between the blocks. Including preparation, the whole experiment lasted approximately 2.5 hours.

### Behavioral and ERP Data Acquisition and Analysis

The E-Prime software program (Psychology Software Tools, Pittsburgh, PA) was used to present the sentences and collect behavioral data. Paired *t* tests were conducted for the comparison between expected and unexpected conditions of both accuracy and reaction time. Only correct trials were included in the ERP and EROS data analysis.

EEG data were recorded using the Neuroscan system. Three channels were used to record at the *Fz*, *Cz*, and *Pz* based on the 10/20 system. Two other electrodes were placed on the left and right mastoid recording sites. The left mastoid site was used as the online reference, and the average of the left and right mastoids was used for the offline re-reference. Another four electrodes were used to monitor the horizontal and vertical EOGs; two were placed on the canthi of the left and right eyes, and the other two were placed above and below the right eye. All electrodes were pasted directly onto the head of the participant. Each electrode was attached to a connecter that could be inserted into the Neuroscan amplifier headbox. The impedance of each channel was kept below 5 KΩ. The EEG data were recorded using a band-pass filter of 0.01 to 100 Hz and sampled at a rate of 250 Hz. Ocular artifacts were corrected using the method provided in Scan 4.3 [33]. The data were then filtered offline using a 0.5 to 30 Hz band-pass filter. The −200 to 1000 ms epoch that was time-locked to the onset of the critical word in each sentence was extracted with the pre-stimulus interval (−200 to 0 ms) as the baseline, and a criterion of ±80 µV was used to reject artifacts.

The peak amplitudes of the N100 (from 50 to 150 ms) and P200 (from 150 to 300 ms) components of the EEG signals were computed for each of the three electrodes, as were the average amplitudes of the N400 (from 250 to 500 ms) and late positive (from 600 to 1000 ms) components. Two-way repeated measures ANOVAs were used to analyze the peak measures and the mean measures separately; the experimental condition (expected vs. unexpected) and electrode location (*Pz* vs. *Cz* vs. *Fz*) were used as factors in the ANOVAs. Greenhouse-Geisser corrections were applied when appropriate.

### EROS Data Acquisition and Analysis

A frequency-domain oximeter (Imagent; ISS, Inc., Champaign, IL) was used for the simultaneous EROS data acquisition during the acquisition of the ERP data. The light sources that were used in obtaining the EROS data were laser diodes emitting light with a wavelength of 830 nm (max amplitude: 10 mW) that was modulated at a frequency of 110 MHz. The light was carried to the surface of the head via individual optic fibers and detected using fiber optic bundles that were connected to photomultiplier tubes (PMTs) that were also positioned on the head. The PMT inputs had a frequency of 110.01000 MHz and generated a 10 KHz heterodyning frequency. The output current from the PMTs was subjected to a fast Fourier transform, which was used to compute the DC (average) intensity, AC amplitude, and relative phase delay (in picoseconds) of the signal.

**Figure 1:**
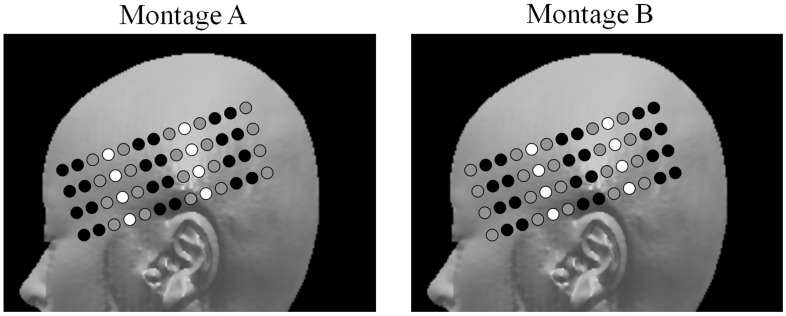
Two montages used in recording the EROS data. The black and white holes represent the locations of the sources and detectors, respectively. The gray holes represent locations that were not used in the montages.

Data were collected from 8 detectors that were coupled to 16 time-multiplexed sources, which resulted in the use of a total of 128 channels in the present study (see Figure 1). The sources and detectors were held against the surface of the left hemisphere of the head using a custom-built helmet. The sampling rate was 31.25 Hz (it took 32 ms to obtain samples from all 16 channels). Following the study of Tse et al. [25], we used two montages in the present study to ensure that we could cover most of the left frontal and temporal gyri (see Figure 1). The order in which the two montages were applied was counterbalanced across participants.

Structural MRI scans were collected for all of the participants. These scans were obtained using a 1.5T Siemens MRI scanner with a standard head coil. High-resolution 3D volume images were acquired (matrix = 256×256, TR = 30 ms, TE = 3 ms, slice thickness = 1.3 mm). Vitamin E pills were placed on the nasion and on the left and right preauricular points of each participant’s head during the MRI scan to aid in the coregistration of the scan and the locations of the optical electrodes. The source and detector locations for both of the montages and for the nasion and left/right preauricular reference points were digitized using the Fastrak 3Space software program (Polhemus Fastrak 3Space®, Colchester, VT). The digitized optical locations were aligned to the surface of the brain using the nasion and preauricular fiducial points and then transformed into Talairach space [34].

The preprocessing of the optical data included the following steps: (a) correcting for phase wrapping and adjusting to a mean of zero for each block; (b) removing the arterial pulse artifact using an algorithm described by Gratton and Corballis [35]; (c) band-pass-filtering the signal (for frequencies of 1 to 10 Hz); (d) extracting the appropriate temporal epoch (−200 to 1000 ms) in which the zero point of the epoch corresponded to the onset of the critical word; (e) averaging the epochs for each time point, condition, channel and participant. The averaged data were analyzed using the OPT-3d software package [36]. To reduce noise, signals from channels with source-detector distances of less than 15 mm or greater than 75 mm were discarded. Channels for which the standard deviation of the phase signal was greater than 210 *ps* were also excluded. Signals from channels that overlapped a given voxel were averaged to increase the signal-to-noise ratio [37].

For the statistic analysis of the EROS data, first we analyzed all signals of the left hemisphere that were covered by the montages (similar to the whole brain fMRI analysis). Specifically, group-level *t* tests were calculated across subjects and were converted to *Z* scores for each voxel at each of the 32-ms time points. The resulting *Z* scores were then orthogonally projected onto images of the sagittal surface of a brain in Talairach space. An 8-mm Gaussian spatial filter was used when generating the *Z* score maps.

Second, the significantly activated brain regions (*p*<0.05) that survived in the group analysis were corrected for multiple comparisons. As the signal-to-noise ratio of the EROS is low, the smaller and restricted ROI (Region Of Interest) method based on random field theory was used to correct for multiple comparisons [38], [39]. Because the activation pattern and the location of the activated regions after group analysis in the present study are consistent with the results from our previous fMRI study using the same paradigm and similar material [31], we selected the ROIs according to the results of our previous fMRI study. These ROIs included the left anterior inferior frontal gyrus (LaIFG, BA47/45), the left posterior inferior frontal gyrus (LpIFG, BA44), the left middle frontal gyrus (LMFG, BA9), the LpMTG (BA22) and the LATL. All of the ROIs except the LATL were 16-mm cubes whose center coordinates were the locations of the peak fMRI values in each of the corresponding brain regions. Given that there is little consensus among various studies regarding the activation of the LATL [40], a larger ROI covering most of the LATL (a 30-mm cube with *x* coordinates from −65 to −35, *y* coordinates from −12 to 18, and *z* coordinates from −30 to −60) was examined for this brain area.

The brain regions we report here are those that reached significance in the group analysis and survived after correction for multiple comparisons.

## Results

### Behavioral and ERP Results

**Figure 2:**
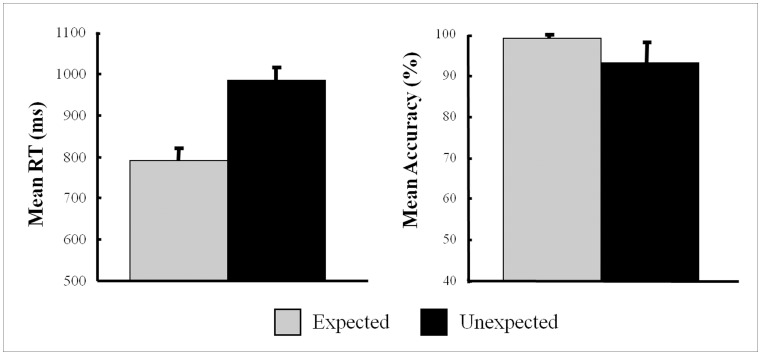
Mean reaction times and accuracies for the two conditions.

As shown in Figure 2, the responses to sentences containing unexpected target words were less accurate (*t*_(14)_ = −4.92, *p*<0.001) and slower (*t*_(14)_ = 9.04, *p*<0.001) than the responses to sentences containing expected target words.

**Figure 3:**
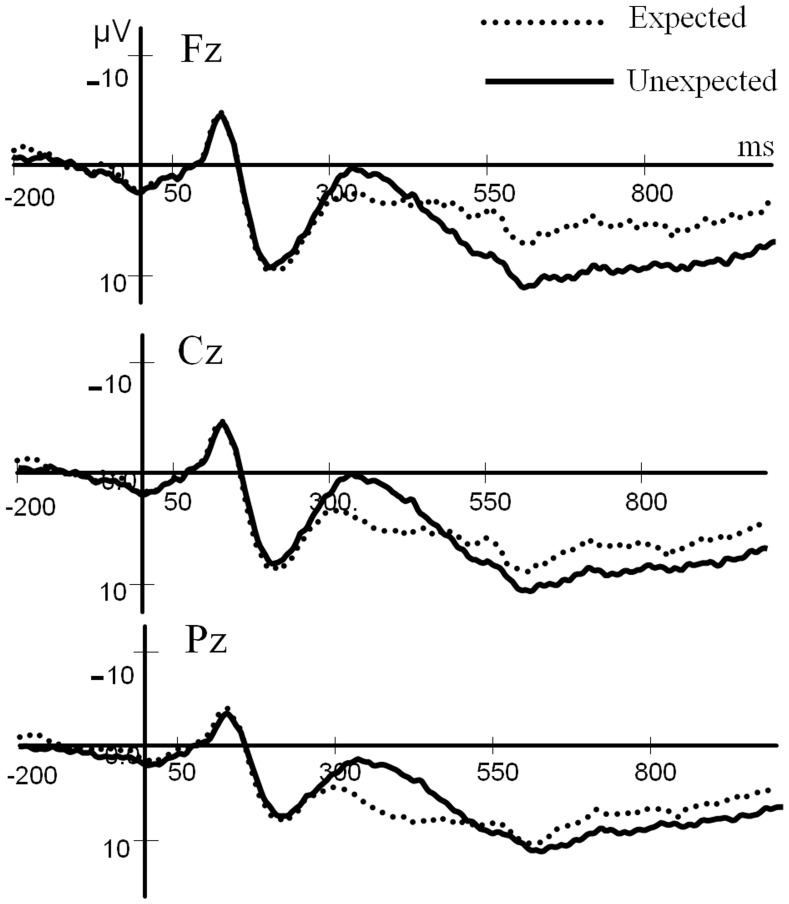
Grand average ERP results for the two conditions.

As illustrated in Figure 3, in an analysis of the peak amplitudes of the N100 components, there was neither a significant main effect of the experimental condition (*F*_(1,14)_ = 0.98, *p* = 0.34) nor an interaction between condition and electrode (*F*_(2,28)_ = 0.69, *p* = 0.51). Similarly, neither the main effect of the experimental condition (*F*_(1,14)_ = 4.49, *p* = 0.06) nor the interaction between condition and electrode (*F*_(2,28)_ = 1.92, *p* = 0.16) was significant in the analysis of the peak amplitude of the P200 component. For the mean amplitude of the N400 component, we did find a significant main effect of condition (*F*_(1,14)_ = 22.84, *p*<0.01) and a significant condition×electrode interaction (*F*_(2,28)_ = 55.01, *p*<0.01). Post hoc comparisons revealed that the unexpected condition elicited a larger N400 than the expected condition at the *Pz* (*p*<0.01) and *Cz* (*p*<0.01) sites; this difference was not apparent at the *Fz* (*p* = 0.1) site, which indicates a canonically distributed N400 effect (the effect on the posterior site was larger than the effect on the anterior site). For the mean amplitude of the late positivity, both the main effect of the experimental condition (*F*_(1,14)_ = 21.19, *p*<0.01) and the condition×electrode interaction (*F*_(2,28)_ = 23.88, *p*<0.01) were significant. Post hoc comparisons revealed that in comparison with the expected condition, the unexpected condition elicited a larger late positivity at the *Pz* (*p*<0.05), *Cz* (*p*<0.01) and *Fz* (*p*<0.01) sites, and the differences toward the front of the head were larger than those toward the back of the head (the effect sizes were 1.29, 2.01 and 3.76 µV for *Pz*, *Cz* and *Fz*, respectively).

### EROS Results

**Figure 4:**
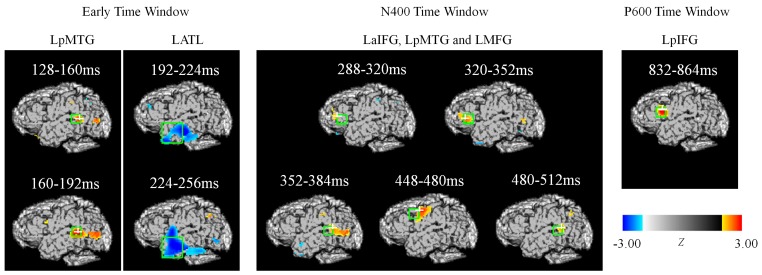
EROS statistical maps of the expectancy effect. *t* tests were conducted and converted to *Z* score maps. The ROI method was used to correct for multiple comparisons. The ROIs (green boxes; 16 mm cubes) were obtained from our previous fMRI data (Huang et al. 2012) with the exception of the LATL ROI (a 35 mm cube). Only the statistically significant results are reported here.

As shown in Table 2 and Figure 4, the LpMTG activation that occurred between 128 and 192 ms was significantly larger in the unexpected condition than in the expected condition. During the N400 time window, greater activation was also observed in the LaIFG (from 288 to 352 ms), the LpMTG (from 352 to 384 ms, and again from 480 to 512 ms) and the LMFG (448 to 480 ms) in response to unexpected words. Finally, the unexpected condition was associated with a larger LpIFG activation than the expected condition during the late positivity temporal epoch (from 832 to 864 ms). Because the LaIFG and the LpIFG are near each other, a jackknife procedure and a *t* test were used to determine whether the locations of these two activation sites differed. The mean location of the LaIFG peak was 20 mm anterior (*t _(14)_* = 4.46, *p*<0.01) and 14.4 mm inferior (*t _(14)_* = 3.86, *p*<0.01) to the location of the LpIFG peak. In addition to the positive activations mentioned above, greater LATL activation in response to expected stimuli (as compared with unexpected stimuli) was observed from 192 to 256 ms after the onset of the target word (negative *Z*-scores indicated in blue in Figure 4).

**Table 2:** Brain regions with significant EROS activation.

Region	Time	*x y z*			Peak *Z*	BA
Unexpected vs. Expected						
LpMTG	128–160ms	−61	−46	7	3.04	BA22
LpMTG	160–192ms	−63	−46	4	3.26	BA22
LaIFG	288–320ms	−52	36	9	2.66	BA45
LaIFG	320–352ms	−51	34	4	2.71	BA45
LpMTG	352–384ms	−60	−48	7	2.46	BA21
LMFG	448–480ms	−50	−1	37	2.54	BA9/6
LpMTG	480–512ms	−57	−48	9	2.45	BA21
LpIFG	832–864ms	−55	14	19	3.29	BA44
Expected vs. Unexpected						
LATL	192–224ms	−37	17	−33	2.99	BA38
LATL	224–256ms	−55	−1	−18	3.06	BA21

Notes. LpMTG, left posterior middle temporal gyrus; LaIFG, left anterior.

inferior frontal gyrus; LMFG, left middle frontal gyrus; LpIFG, left posterior.

inferior frontal gyrus; LATL, left anterior temporal lobe; *x y z*, Talairach coordinates; BA, Brodmann’s areas.

## Discussion

Consistent with previous ERP studies [30], [41], [42], we found that unexpected words evoked a larger N400 and a larger late positivity than expected words. Moreover, our EROS experiment replicated the activation patterns in most brain regions related to semantic processing reported by previous fMRI studies [31], [43]. Specifically, we observed stronger activation in the LpMTG, the LaIFG, the LpIFG, and the LMFG for unexpected words than for expected words. However, whereas few previous studies on sentence comprehension have reported activation in the LATL, we found that this region was more strongly activated by expected words than by unexpected words. More importantly, using the EROS technique, we captured the temporal dynamics of semantic processing in the above-mentioned brain regions. Below, we will discuss the activation patterns occurring during different time windows.

### Lexical-semantic Retrieval during the Early Time Window

Our EROS data revealed stronger activation in the LpMTG between 128 to 192 ms for unexpected words than for expected words. This finding is consistent with the result of a previous EROS experiment showing greater LpMTG activation for incongruent target words than for congruent target words during a similar time window [25]. It should be noted that the LpMTG is generally believed to be involved in lexical-semantic retrieval [4], [14], [15], [16]. As the unexpected words in our experiment did not fit the prior context, participants might have needed to recruit more resources to retrieve the meanings of these words, which in turn led to the stronger LpMTG activation that we observed [31].

Tse et al. [25] also observed activation in the LpMTG in the early time window, although the onset reported in that study (from 179 ms) was slightly later than that observed in our study (from 128 ms). This difference may be due to the different paradigms used in the two studies. Specifically, unlike in the study by Tse et al. all of the target words in our study were preceded by highly constraining context, so participants could rapidly realize the mismatch between the target word and the context, and allocate more cognitive processes to retrieve the proper semantic information about the target words [31], [44]. This in turn would be reflected by the early activation of the LpMTG. Despite the slight difference in the onset of the LpMTG activation, both studies showed that the LpMTG is activated in the very early time window, providing evidence that lexical-semantic retrieval can occur very early, rather than during the N400 time window [45].

It is worth mentioning that although the EROS data in both the present study and Tse et al. [25] revealed the LpMTG activation in the early time window, no early ERP effects were found in either study, which is in line with the findings of most previous studies on sentence processing. The lack of any early ERP effects, according to some researchers, may be because early semantic effects could easily be obscured, especially because early ERP components are typically focal, short in duration, and highly sensitive to the physical characteristics of stimuli (e.g., word frequency and length). In psycholinguistic studies, to improve ecological validity, researchers often select materials with a large range of physical features. However, stimuli with physical features of different ranges (e.g., high- vs. low-frequency words) would elicit ERP components of different shapes, which in turn may obscure any early effects [9].

### Semantic Integration in the LATL: A Relatively Rapid and Transient Response

During a slightly later time window from 192 to 256 ms, our EROS data revealed stronger activation in the LATL for expected words than for unexpected words. The LATL activation has also been reported by some previous studies on sentence processing, and this region has traditionally been thought to reflect integrative syntactic processing [13]. For example, compared with random word lists, normal sentences triggered larger activation in the LATL [19], [46]–[51]. Moreover, greater activation was observed in the LATL for syntactically reasonable sentences with content words replaced by pseudowords when compared with meaningless pseudoword lists [17], [50]. Furthermore, stronger activation in the LATL was found for structurally complex sentences relative to simple sentences [52]–[54] but no difference in Stowe et al. [49]; Friederici et al. [55]. According to a recent MEG study, phrases that could be integrated syntactically evoked greater activation in the LATL between 184 and 255 ms than phrases that could not be integrated [56], suggesting that syntactic integration occurs rapidly and automatically in this region. Nevertheless, the LATL has recently been shown to be related to semantic processing in studies on semantic memory, semantic priming, and semantic processing during sentence and discourse comprehension. For example, studies on semantic memory suggested that the LATL acted as the semantic hub for the integration of various surface features into abstract semantic representation in the conceptual system [57]. Moreover, studies on semantic priming found that the unprimed condition triggered larger activation in the LATL than did the primed condition [58]–[64]. In addition, studies on sentence comprehension found that relative to sentences composed of pseudowords or words in a random order, semantically congruent sentences elicited larger activation in the LATL [17], [19]. Furthermore, Rogalsky and Hickok [18] reported a sentence-level study in which participants were required to finish a semantic or a syntactic task for the same sentences. They found that the LATL was activated in both tasks. Similarly, in studies on discourse comprehension, researchers have found that compared with incoherent discourse, coherent discourse evoked larger activation in the LATL [65].

As we did not manipulate any syntactic variables in the present study, but found greater activation in the LATL for sentences that could be integrated easily at the semantic level, we prefer to interpret this activation as reflecting integrative semantic processing, through which participants constructed coherent sentence representations. Note that the unexpected and expected conditions in our experiment differ in both the difficulty of semantic integration and the ease of retrieving the meanings of the target words as a function of semantic priming differences caused by contextual constraints [66]. However, we believe that the observed activation pattern reflects semantic integration but not lexical-semantic retrieval because the LATL activation was stronger for the expected condition than for the unexpected condition, opposite to the pattern evoked by semantic priming.

Furthermore, the finding that greater LATL activation in the expected condition was found in the very early time window (from 192 to 256 ms) suggests that the semantic integration that happened in this region may be relatively rapid and transient. Interestingly, the time window of the LATL activation we found is very similar to that observed for syntactic processing in approximately the same region [56], which is in line with the idea that this area may index some rapid and possibly automatic processes of integration at both semantic and syntactic levels [18]. Further studies may be required to explore the possibility that different sub-regions of the LATL are selectively responsible for semantic and/or syntactic integration [17], [18].

One might question why, if the LATL indeed plays a vital role in semantic processing, previous fMRI studies have rarely reported activation in this region. Two possibilities may account for this. First, the LATL is located near the air-filled cavities, where fMRI signals are easily distorted and thus cannot sensitively detect the activation in this area [40], [67]. In addition, most studies using the violation and expectancy paradigm have often focused on the larger brain activations evoked by the violated or unexpected condition rather than on those evoked by normal sentences. In such studies, the larger LATL activation caused by congruent sentences may be neglected.

It is worth noting that if the early LATL reflects semantic integration, the LATL activation should have been observed in both the expected and the unexpected conditions. Moreover, the peak of the LATL activation should have been later for the unexpected than for the expected targets, because the integration of the unexpected ones presumably takes longer. However, we could not directly verify these possibilities in the present study. This is because the current design did not include a baseline minimally engaging the LATL, such as the arithmetic condition [68], that can be used to evaluate the absolute activation levels and the activation peaks of the LATL of the expected and unexpected conditions. Further studies may be required to explore the above-mentioned possibilities.

### Semantic Integration in the LIFG: A Relatively Slow but Enduring Response

During the 250 to 500 ms time window, unexpected words elicited a larger N400 than did expected words. Moreover, the corresponding EROS results revealed stronger activation in the LaIFG, the LpMTG and the LMFG for unexpected words than for expected words. These findings are consistent with previous MEG studies on sentence reading which found stronger activation in the temporal lobe [69]–[73] and the frontal lobe [74]–[77] for the semantically violated words than the congruent ones in the N400 time window. Moreover, a recent MEG study suggested that both the LIFG and the LMTG could be the source of the semantic N400 effect [77]. Altogether, the available evidence appears to suggest that the LIFG and the LMTG interact dynamically to perform semantic processing during the N400 time window. But the current study further suggests that only the anterior of the LIFG but not the whole LIFG involves in semantic processing in the N400 time window.

Previous fMRI studies have implicated the LaIFG in sentence-level semantic integration [13], [15], [78] and the LpMTG in lexical-semantic retrieval, which may explain our results. Specifically, our participants might resort to top-down processes to integrate unexpected words into the prior context, which may lead to stronger LaIFG activation [15]. During this integration process, retrieval and maintenance of the proper meanings of unexpected words would be necessary, which may cause greater LpMTG activation [79]. Contrary to the “rapid” integration associated with the early LATL activation described above, the integration during the N400 window takes longer and involves multiple brain regions. We therefore refer to this as a relatively “slow” and enduring integration.

In addition to the activation in the LaIFG and the LpMTG, we also observed LMFG activation during the N400 time window. The LMFG is not typically associated with semantic processing in alphabetic languages, but it has been shown to be related to semantic processing at the word, phrase, and sentence levels in previous studies on Chinese [80]–[87]. Our results further suggest that the LMFG may play a unique role in the relatively slow and enduring integration during sentence comprehension in logographic Chinese.

### Language Comprehension and Cognitive Control

Consistent with previous studies using similar designs, we observed a larger late positivity for unexpected words than for expected words from approximately 600 ms after target word onset [41], [42], [88]. Traditionally, the late positivity has been associated with syntactic processing [13], [89], but recent studies have shown that semantic variables could also elicit this response [90], [91]. Some researchers suggest that this component may reflect the reassignment of thematic roles in response to animacy violations [90] that engages a frontal/inferior parietal/basal ganglia network [92] However, other researchers propose that the late positivity may reflect a domain-general processing mechanism, such as cognitive control or conflict detection [93]–[95], that usually involves the LIFG [96], [97]. Since our experimental items did not contain animacy violations, the late positivity we observed should not be attributed to the reassignment of thematic roles, but may instead reflect more general cognitive processing.

The EROS data showed that the LpIFG was activated during the 832 to 864 ms time window. Previous fMRI studies have suggested that the LIFG can be subdivided into two regions with different functions: the LaIFG and the LpIFG. Specifically, the former appears to respond to semantic integration, and the latter to engage in general cognitive processing [43]. Our finding that the LaIFG and the LpIFG were activated during the N400 and the late positivity time windows, respectively, provides further evidence for the idea that these two subregions function at different stages of semantic processing. Thus, general cognitive processing may exert an influence at a relatively late stage of semantic processing, assisting readers to accomplish the specific experimental task used in this study.

### Conclusion

The most important contribution of this study is that we revealed the dynamic temporal order among various brain regions related to semantic processing when they work together to achieve incremental sentence comprehension. Specifically, the LpMTG, which is responsible for lexical-semantic retrieval, is activated as early as 200 ms after stimulus onset. Following this LpMTG activation are two semantic integration processes. The first, a relatively “rapid” and transient integration indicated by the LATL activation approximately 200 to 300 ms after stimulus onset, is generally associated with the processing of congruent sentences. The second, a relatively “slow” and enduring integration as reflected by the activation in the LaIFG, the LpMTG, and the LMFG during the N400 time window (250 to 500 ms after stimulus onset), appears to be related to the processing of sentences containing unexpected or violated words. During an even later time window, general cognitive control indexed by the LpIFG activation becomes involved, enabling participants to finish the specific experimental task. To conclude, the results obtained in the present study suggest that during sentence comprehension, lexical-semantic retrieval occurs first, followed by the interaction between lexical-semantic retrieval and semantic integration in the N400 time window.
